# Multiscale investigation of graphene layers on 6H-SiC(000-1)

**DOI:** 10.1186/1556-276X-6-171

**Published:** 2011-02-24

**Authors:** Antoine Tiberj, Jean-Roch Huntzinger, Jean Camassel, Fanny Hiebel, Ather Mahmood, Pierre Mallet, Cecile Naud, Jean-Yves Veuillen

**Affiliations:** 1 Groupe d'Etude des Semiconducteurs, UMR5650 CNRS-Université Montpellier II, cc074, Place Eugène Bataillon, 34095 Montpellier Cedex 5, France; 2 Institut Néel, CNRS-UJF, Boîte Postale 166, 38042 Grenoble Cedex 9, France

## Abstract

In this article, a multiscale investigation of few graphene layers grown on 6H-SiC(000-1) under ultrahigh vacuum (UHV) conditions is presented. At 100-μm scale, the authors show that the UHV growth yields few layer graphene (FLG) with an average thickness given by Auger spectroscopy between 1 and 2 graphene planes. At the same scale, electron diffraction reveals a significant rotational disorder between the first graphene layer and the SiC surface, although well-defined preferred orientations exist. This is confirmed at the nanometer scale by scanning tunneling microscopy (STM). Finally, STM (at the nm scale) and Raman spectroscopy (at the μm scale) show that the FLG stacking is turbostratic, and that the domain size of the crystallites ranges from 10 to 100 nm. The most striking result is that the FLGs experience a strong compressive stress that is seldom observed for graphene grown on the C face of SiC substrates.

## Introduction

The unique electronic, optical, and mechanical properties of graphene [1-3] give rise to an intense research activity for both scientific and technological purposes. Among these research activities, special effort is devoted to develop preparation techniques [4-12] which yield large-scale graphene wafers of high quality and uniformity. Today, one of the most promising methods for microelectronic applications consists in a controlled sublimation of a few Si atomic layers from a single crystalline SiC surface [9-20]. The remaining C atoms rearrange themselves and form few layer graphene (FLG), often called "epitaxial graphene." Such FLG samples can be grown either on the Si face (0001) of a SiC substrate or on the C face (000-1). Graphene growth on the Si face has been extensively studied in the last few years [9-12]. It has been shown that large, homogeneous graphene monolayers and bilayers can be obtained on top of a $6\sqrt{3}\times 6\sqrt{3}R30$ SiC surface reconstruction [13-15]. The graphene planes are Bernal (AB) stacked. The interface between the first graphene plane and the SiC surface is composed of an intermediate C-rich layer having covalent bonds with Si atoms of the substrate [13-15]. Epitaxial graphene on the Si-face is usually highly *n*-type doped (around 10^13 ^cm^-2^) with a low carrier mobility (usually few thousands cm^2 ^V^-1 ^s^-1^).

On the C face, the situation is completely different. There is no need for a buffer layer anymore but two different pristine surface reconstructions exist below the graphene layers: (2 × 2)_C _and (3 × 3) SiC reconstructions, and the graphene layers have several orientations on top of each surface reconstruction [16-18]. The interaction between graphene layers and the C face of SiC substrate is reduced compared to the one existing on the Si face. Graphene grown on (3 × 3) SiC surfaces experience the weakest interaction with the underlying substrate. This weaker interaction between the graphene layers and the SiC substrate may be one of the reason for the better carrier mobility measured on epitaxial graphene on C-face (27000 cm^2 ^V^-1 ^s^-1^) [19]. It also explains why, long, self-ordered, strain-free graphene ribbons can be grown on large reconstructed terraces [20]. Besides, it has been shown that the interaction with the environment impacts also the transport properties of exfoliated graphene [21]. It is therefore of primary importance to study the graphene/SiC interaction. The focus of this article will be on FLG grown in ultrahigh vacuum (UHV) on 6H-SiC (000-1). Surface reconstruction will be probed by low-energy electron diffraction (LEED), Auger electron spectroscopy (AES), and scanning tunneling microscopy (STM). Thanks to previous studies [17,18], SiC surface reconstruction, graphene orientation, and stacking can be determined from the Moiré patterns observed in STM. The observed crystallite size and stacking will be compared to those from Raman spectroscopy performed on the same sample. Despite the different sizes of the probed area between STM (microscopic) and Raman spectroscopy (macroscopic), a very good agreement has been found.

### Graphene growth

**Figure 1:**
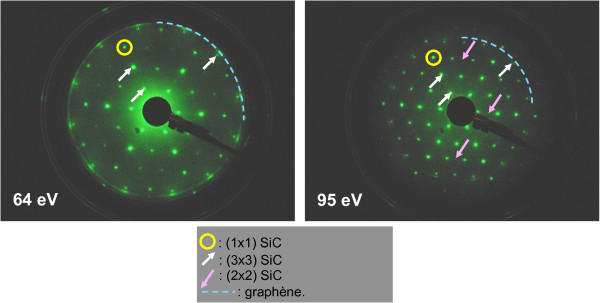
**LEED patterns of a 6H-SiC(000-1) sample after graphitization**. The circles indicate the (1 × 1) SiC spots, the white arrows the (3 × 3) SiC spots, and the rose arrows point to faint SiC(2 × 2)_C _spots. The dashed (quarter) circle shows the ringlike pattern of graphene. The modulated intensity of FLGs signal corresponds to the distribution of rotation angles for the first graphene layer compared to the SiC surface with some preferential orientations (the brightest spots).

The sample graphitization and first characterization (STM, LEED, and AES) were performed *in situ *under UHV conditions according to the procedure of ref. [18]. The surface of the 6H-SiC(000-1) sample (n doped, purchased from NovaSiC) was first cleaned by a 850°C annealing treatment under a Si flux. The usual SiC(3 × 3) reconstruction [16] was obtained by further heating at 950-1000°C. Graphitization of the surface was performed by annealing at increased power until a graphitic signal is detected by LEED. The typical diffraction patterns (shown in Figure 1) reveal both SiC(3 × 3) and SiC(2 × 2) spots indicated by arrows with SiC(1 × 1) spots being indicated by circles. The FLGs exhibit a ringlike LEED pattern with modulated intensity indicated by the dashed circle. It evidences a significant distribution of azimuthal disorientation for the first graphene plane compared to the SiC lattice. However, some preferential orientations exist, as shown by the more intense spots (at 30° and predominantly around 14°). AES performed on this sample gives an average coverage of the surface of 1 to 2 graphene layers.

### Scanning tunneling microscopy

**Figure 2:**
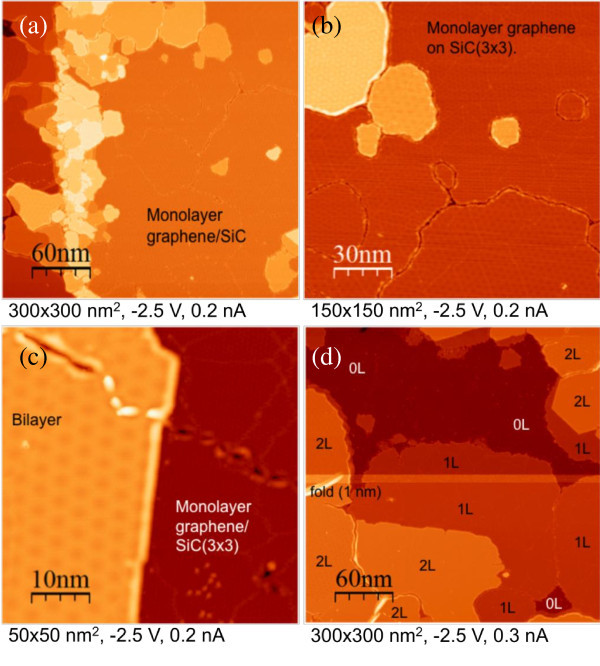
**STM images of a 6H-SiC(000-1) sample after graphitization (same sample as in figure 1)**. **(a) **300 × 300 nm^2 ^STM image of few layers of graphene grown on 6H-SiC (000-1). The brightest areas correspond to multilayers grown on a step edge, the right part corresponds to monolayer graphene. **(b) **150 × 150 nm^2 ^zoom of the top right corner of image **(a)**. The dark areas correspond to monolayer graphene grown on (3 × 3) SiC-reconstructed surface. MPs are seen on the monolayer and on the multilayers (the brighter area) indicating that the first graphene layer is disoriented compared to the SiC surface and that the multilayers are "twisted" (turbostratic stacking). **(c) **50 × 50 nm^2 ^STM images of a bilayer and a monolayer. The turbostratic stacking of the bilayer is revealed by a long-range MP with a wavelength of 4 nm. On the monolayer, one can only see the (3 × 3) SiC surface reconstruction pattern due to the high tunnel voltage (-2.5 V). It should be stressed that the top graphene plane is continuous between the mono and the bilayer. **(d) **300 × 300 nm^2 ^STM image showing the distribution of FLG grown ranging from the bare (3 × 3) SiC surface (0L), monolayers (1L), and bilayers (2L). On the left, a 1-nm high wrinkle can be seen on a bilayer. The bright horizontal line corresponds to a tip change.

STM measurements were done at room temperature using mechanically cut PtIr tips. Typical STM images are gathered in Figure 2 in which a large diversity of graphene layers can be observed. First, on the edge of the SiC reconstructed steps, the growth rate is much higher, and small multilayers which are a few tens of nm width appear (Figure 2a). On the terraces, mono and bilayers cover the majority of the surface and are much wider (up to 100 nm). Few small areas are not graphitized, and the usual SiC(3 × 3) surface reconstruction can be observed (Figure 2d) [16]. The (3 × 3) is also seen on Figure 2c through the graphene monolayer because of the high sample bias (-2.5 V) [17,18]. In Figure 2b,c one can also clearly distinguish some Moiré patterns (MP) on graphene mono, bi, and multilayers. Such MPs have several origins. The MP observed on the monolayer graphene comes from disorientation between the first graphene plane and the SiC(3 × 3) surface. The disorientation angle determines the period of the MP based on a classical model previously described [18]. For instance, the MP for the island in the lower right part of Figure 2b corresponds to a rotation angle of 11.2°. The MPs observed on the multilayers come both from the interface (as above) and from rotational stacking faults between the different graphene planes, which is characteristic of a turbostratic stacking. Such disorientations between the graphene sheets and the SiC substrate confirm the weak coupling between the graphene planes, and also between the FLGs and the SiC substrate. This is corroborated by the presence of wrinkles seen in Figure 2d. Finally, the top graphene plane on Figure 2c is a continuous sheet between the mono and the bilayer graphenes.

### Raman spectroscopy

**Figure 3:**
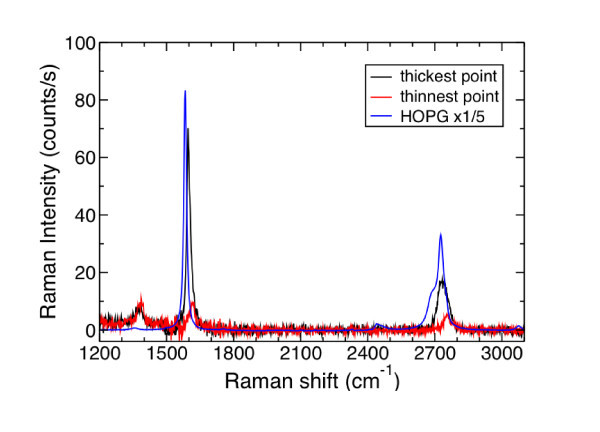
**Raman spectra of a HOPG sample (blue line), the thickest FLG (black line), and the thinnest FLG (red line)**. The FLG's spectra are extracted from the 16 × 16 μm^2 ^Raman mapping collected in the center of the sample. D band can be seen around 1350 cm^-1 ^indicating the presence of crystalline defects/disorder in the grown FLG. The 2D band around 2750 cm^-1 ^has a single Lorentzian shape that is the fingerprint of a turbostratic stacking. Finally, both G and 2D bands are shifted to high energy compared to the HOPG spectrum. This up shift evidences that FLGs experience a high compressive stress.

**Figure 4:**
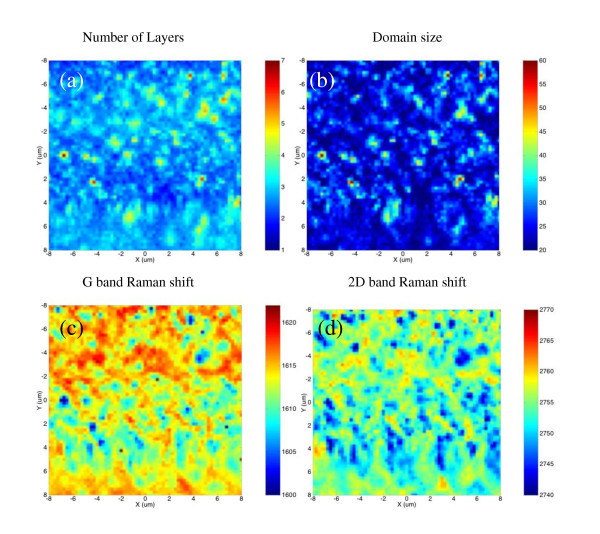
**16 × 16 μm**^**2 **^**Raman maps collected with a 0.25-μm step size**. **(a) **FLG thickness derived from the normalized integrated intensity of the G band. The thickness is comprised between two and six graphene planes with an average thickness of three planes. **(b) **Domain size of the graphene crystallites deduced from the *I*_D_/*I*_G _ratio. The in-plane size ranges from 20 to 60 nm. **(c, d) **Raman shift of the G and 2D bands, respectively. The positions of both bands are shifted to higher energies. The G band is around 1610 cm^-1 ^and the 2D band around 2750 cm^-1^. This high up shift can only be explained by a high in-plane compressive strain of the graphene lattice.

To investigate the quality and thickness uniformity of the FLG, micro-Raman spectroscopy and microtransmission measurements were simultaneously performed. As has already been shown [20], these two techniques can be easily combined by inserting a low-noise photodiode between the SiC substrate and the *XYZ *piezoelectric stage. It is then possible to measure at the same time, during the acquisition of Raman spectra, using the same laser beam as a probe, the power transmitted through the sample. Raman spectra were collected at room temperature using a Jobin Yvon-Horiba T64000 spectrometer in the confocal mode, with a ×100 microscope objective. The 514-nm line of an Ar ion-laser was used for excitation. The spot size was 1 μm, with 1-mW incident power under the objective. Using this original combination of techniques, a 16 × 16 μm^2 ^mapping of the FLG area located at the center of the sample was performed. The step size was 0.25 μm along both *X *and *Y *directions. Since no bare SiC surface could be found at the probe size, a SiC reference spectrum was collected by focusing the laser beam in the SiC substrate deeper than the confocal field depth. FLG's Raman spectra were obtained by subtracting the SiC reference spectrum from the experimental spectra. Typical spectra, collected on the thinnest and thickest FLG parts, are compared to the one of a highly oriented pyrolytic graphite (HOPG) sample in Figure 3. On these spectra, D, G, and 2D bands can easily be observed at 1380, 1610, and 2750 cm^-1^, respectively. These three bands are blue shifted compared with standard FLG and HOPG Raman spectra. As discussed later, this blueshift can only be explained by a high compressive strain of the graphene lattice. The 2D band has a single Lorentzian shape meaning that the FLG stacking is not Bernal but, rather, turbostratic. This first observation is in perfect agreement with the previous STM results. The D band around 1380 cm^-1 ^comes from the breakdown of the wavevector selection rule and reveals the presence of crystalline defects inside or at the edges of FLG flakes. The in-plane size of the crystallites can be deduced from the ratio between the G and D band integrated intensities (*I*_D_/*I*_G_). Using the expression given by Pimenta et al. [22] the domain size map was extracted, as shown in Figure 4b.

(1) $${\left(L_{\text{a}}\right)}_{\text{nm}}=2.4\times 10^{-10}{\left(\lambda_{\text{laser}}\right)}_{\text{nm}}^{4}{\left(\frac{I_{\text{D}}}{I_{\text{G}}}\right)}^{-1}$$

The in-plane sizes of the crystallites *L*_a _ranges from 20 to 60 nm, which are in excellent agreement with previous STM observations.

To estimate the average graphene thickness, one should use the relative extinction deduced from our microtransmission measurements [20]. Unfortunately, on this sample, no bare SiC substrate could be measured. It was then impossible to measure the transmitted power through the SiC substrate. Hopefully, from previous results [20] it is also known that the thickness can be roughly estimated from the G band-integrated intensity. In this case, for thin FLG (less than five layers), the estimated error is one layer, while, for thicker samples, the estimated thickness may have a factor two error. The following (empirical) relationship was used, which corresponds to the experimental configuration of this study:

(2) $$n=\frac{I_{\text{G}}}{I_{\text{G HOPG}}}\times \frac{1}{0.03}$$

Laser power fluctuations are corrected thanks to an additional low-noise photodiode that measures the laser power during the acquisition of the Raman map, and the estimated thickness found is shown in Figure 4a. At the scale of the Raman probe, the FLG coverage ranges from two to seven graphene planes, with an average of two to three graphene planes. This is one monolayer thicker than the value deduced from AES and STM experiments. Such discrepancy occurs because of the different sizes of areas probed by Raman spectroscopy (1 μm), AES (100 μm), and STM (few nm up to 300 nm). Moreover, STM focuses always on the most interesting area of the sample (i.e., the thinnest FLG) where bare SiC surface, mono, and few layers can be measured. However, it has already been shown in Figure 2a,b that thick multilayer flakes grow close to the edges of SiC steps. This is not peculiar to these images. The growth rate is always higher at step edges. It can also be stated from Figure 4a that the thickest FLGs measured by Raman spectroscopy are located at discrete spots with a small lateral extension (< 2 μm). This is the case of multilayers seen in Figure 2a, while the majority of the probed area corresponds to FLG with less than three graphene planes. As previously said, using only the G band-integrated intensity, one can have an error of one graphene plane. The thickness estimated from Raman mapping is then consistent with STM results. Concerning AES, the agreement is poorer, and it would be interesting to perform a more detailed cross calibration of AES vs Raman spectroscopy to better understand the correlation between these two different techniques.

Finally, the most striking result is the strong blueshift observed on this FLG. The D band lies around 1380 cm^-1^, the G band around 1610 cm^-1^, and the 2D band around 2750 cm^-1 ^whereas neutral relaxed graphene has a D band around 1350 cm^-1^, a G band centered at 1582 cm^-1^, and a 2D band at 2690 cm^-1^. The average up shift is then of 28 cm^-1 ^for the G band, and of 60 (30) cm^-1 ^for the 2D (D) band, respectively. The G and 2D band Raman shift maps are shown in Figure 4c,d. The G band Raman shift ranges from 1598 to 1626 cm^-1 ^and the 2D band from 2736 to 2764 cm^-1^. Such high blueshift cannot be explained by a doping of the graphene layers. Although high *p*-type doping (3 × 10^13 ^cm^-2^) and *n*-type doping (4 × 10^13 ^cm^-2^) induce a blueshift of the G band up to 1610 cm^-1^, the G band is then narrowed with a FWHM around 8 cm^-1 ^[23]. In this case the FWHM of the G band ranges from 15 to 35 cm^-1^. This G band broadening refutes the high doping hypothesis. The doping hypothesis is also refuted by the 2D band position that cannot be explained by the *n*-type doping that shifts the 2D band to lower energies (down to 2660 cm^-1^) and by the *p*-type doping that shifts the 2D band up to 2700 cm^-1^, which is much smaller than the observed shifts. These blueshifts can actually be explained by a high compressive strain of the graphene lattice. This strain must originate from the strong difference in the in-plane thermal expansion coefficients of the SiC and the graphene. It is created during the cooling down of the sample after the growth. It can be assumed that it is biaxial. The strain and stress can then be deduced from the relationships of Table 1[3,24]. The frequency shifts of the G and 2D are given for a biaxial strain of 1% and for a biaxial stress of 1 GPa.

**Table 1:** Frequency shifts of the G and 2D bands for a biaxial strain of 1% or a biaxial stress of 1 GPa [3,24].

$\varepsilon_{\text{biax}}=1\%$	$\sigma_{\text{biax}}=1\text{ GPa}$
$\Delta \omega_{\text{G}}^{\text{biax}}=-60{\text{ cm}}^{-\text{1}}$	$\Delta \omega_{\text{G}}^{\text{biax}}=-4.8\;{\text{cm}}^{-\text{1}}$
$\Delta \omega_{2\text{D}}^{\text{biax}}=-153{\text{ cm}}^{-\text{1}}$	$\Delta \omega_{\text{G}}^{\text{biax}}=-12.3\;{\text{cm}}^{-\text{1}}$

**Figure 5:**
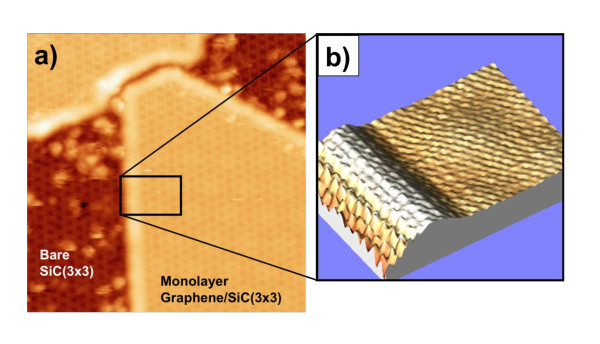
**STM images of the edges of the graphene islands**. **(a) **23 × 23 nm^2 ^STM image of a monolayer on top of the (3 × 3) SiC-reconstructed surface. This image was recorded at a high tunnel voltage (-2.5 V) with a current of 0.2 nA. With such high voltage, only the (3 × 3) SiC surface can be imaged on the bare SiC and through the monolayer. One can still distinguish that the edges of the monolayer are brighter than the center. It corresponds to a height difference of 40 pm. **(b) **To get a more detailed image of the edges, a 6.6 × 4.6 nm^2 ^STM image was recorded at low tunnel voltage (+10 mV) with a current of 0.1 nA. In these experimental conditions, the graphene lattice can be probed with an atomic resolution. The graphene edge is folded and bent towards the SiC surface. The height of the fold is 50 pm. This particular shape is consistent with the compressive stress deduced from the Raman spectra. This compressive stress is occured by the thermal stress because of the cooling down of the sample after the growth and because the graphene edges seem to be bound to the SiC surface.

Using these relationships, it can be estimated from the G band that the strain ranges from -0.2 to -0.7% with an average of -0.5%. The corresponding stress values range from -3 to -8.7 GPa with an average of -6 GPa. From the 2D band, a strain comprised between -0.3 and -0.5% with an average of -0.4% is found. The corresponding stress ranges from -3.7 up to -6 GPa with an average of -5 GPa. The strain/stress derived from the 2D band is slightly smaller than the one deduced from the G band. It can arise from the uncertainty on the graphene/graphite Grüneisen parameters [3] and maybe from a small FLG doping that can induce a small shift of both bands. Nevertheless, it can be concluded from this high up shift of the D, G, and 2D bands that FLG are subjected to a high compressive strain (stress) with an average from around -0.4 to -0.5% (-5 to -6 GPa). The most probable origin is the thermal stress due to the cooling down of the sample after the growth. This compressive stress might be considered as being in contradiction with the weak interaction between graphene layers and the underlying substrate that was shown by LEED and STM experiments. The most likely hypothesis is that graphene crystallite edges are bound to the SiC surface. This bonding would induce this strong thermal stress, but let graphene layers free to grow with the wide distribution of disorientation angles revealed by STM and LEED results. To check this hypothesis, STM images of the graphene edges were recorded and are shown in Figure 5. Unfortunately, the SiC surface and the graphene lattice cannot be imaged simultaneously since graphene is only clearly resolved at low bias within the gap of the SiC(3 × 3) surface [18]. In Figure 5a, the SiC surface is probed at high tunnel voltage (-2.5 V), and the bare (3 × 3) SiC surface can be distinguished from the (3 × 3) SiC surface with a graphene monolayer on top. It can also be stated that the edges of the graphene island are higher than the core by 40 pm. At lower tunnel voltage (+10 mV), a detailed image of the graphene edges was recorded with atomic resolution on the graphene lattice. On this image, it can be clearly observed that the graphene sheet is folded and that its edges are bent towards the SiC surface. The fold is 50 pm higher than the island core at this low bias, which is similar to the high bias value and thus should reflect the topography of the graphene edge. This step height is much smaller than the wrinkles that are usually seen on graphene grown on the C face. However, the fold and the bending can indicate that the graphene edges are bound to the SiC substrate. This edge bonding can also explain the thermal compressive stress of these graphene crystallites after cooling down the sample. Usually on the C face, the elastic energy associated with the stress is often relaxed by the formation of wrinkles. It is not the case here because of the small graphene crystallite sizes. The authors observed indeed only one wrinkle on Figure 2d. The compressive stress is not relaxed because the elastic energy to be relaxed is proportional to the domain area and is not high enough to compensate the energy cost of the wrinkle formation.

## Conclusion

The FLG has been grown on 6H-SiC (000-1) in UHV conditions. An average thickness of one to two graphene planes was found from AES, whereas Raman spectroscopy results indicate an average thickness of two to three graphene planes. This small discrepancy might occur because of the uncertainty of one to two layers for thickness determined from Raman spectroscopy and from the difference of the spot size between these two techniques. LEED and STM experiments show (i) a (3 × 3) SiC surface reconstruction, (ii) a wide distribution of disorientation between the first graphene sheet and the SiC surface, and (iii) rotational stacking fault between the graphene layers corresponding to a turbostratic stacking for the multilayers. The single Lorentzian shape of the 2D Raman band measured on these FLG confirms this turbostratic stacking. The FLG domain size (deduced from the *I*_D_/*I*_G _ratio) ranges from 20 to 60 nm, which is in excellent agreement with the graphene crystallites size probed by STM. Finally, the most striking result is that the D, G, and 2D bands are highly blue shifted (+30, +28, and +60 cm^-1^, respectively). This means that the graphene lattice is highly compressively strained (around -0.4/-0.5%). Usually, FLG grown on the C face of SiC are fully relaxed by forming wrinkles to release the thermal stress during the cooling down of the sample after the growth. For this particular case, the strain might arise because graphene crystallite edges are bound to the SiC surface.

## Abbreviations

AES: Auger electron spectroscopy; FLG: few layer graphene; HOPG: highly oriented pyrolytic graphite; LEED: low energy electron diffraction; STM: scanning tunneling microscopy; UHV: ultrahigh vacuum.

## Competing interests

The authors declare that they have no competing interests.

## Authors' contributions

FH and AM prepared the samples and carried out the LEED, Auger and STM measurements. FH and PM performed the analysis of the LEED, Auger and STM data. AT and JRH carried out the micro Raman spectroscopy and microtransmission experiments. AT, JRH and JC performed the analysis of the Raman and transmission data. AT drafted the manuscript. JC, JRH, CN and JYV participated in the writing of the manuscript. All authors read and approved the final manuscript.
